# Application of antibiotics for the selective isolation of previously uncultured species from activated sludge

**DOI:** 10.1128/spectrum.01477-26

**Published:** 2026-06-22

**Authors:** Stefania Andrea Rosso Villanelo, Sofie Zacho Vestergaard, Lei Liu, Yu Yang, Inge Søkilde Pedersen, Per Halkjær Nielsen, Morten Kam Dahl Dueholm

**Affiliations:** 1 Center for Microbial Communities, Department of Chemistry and Bioscience, Aalborg University, Aalborg, Denmark; 2 Department of Clinical Medicine, Aalborg University 1004, Aalborg, Denmark; 3 Department of Molecular Diagnostics, Aalborg University Hospital 53141, Aalborg, Denmark; Connecticut Agricultural Experiment Station, New Haven, Connecticut, USA

**Keywords:** cultureomics, antibiotics, activated sludge

## Abstract

The microbial communities in activated sludge (AS) drive pollutant degradation and nutrient transformation into biomass and gaseous products, while also enabling resource recovery processes. In these systems, microorganisms grow as flocs, whose aggregation properties are essential for retaining active biomass while producing a clarified effluent. Understanding the microbial composition of AS and the functions of individual taxa is crucial for improving wastewater treatment practices and developing new treatment technologies. Although DNA-based studies have identified abundant taxa and inferred their metabolic roles, many of these organisms remain uncultured, limiting experimental validation of genome-based predictions. Here, we investigated whether antibiotics can transiently reduce community complexity and alleviate competitive exclusion during cultivation, thereby facilitating isolation of previously uncultured activated sludge bacteria. Dispersed single cells from AS were cultivated on agarose plates containing filter-sterilized AS fluid and 1 of 11 antibiotics at three concentrations. Full-length 16S rRNA gene amplicon sequencing indicated that antibiotics reduced microbial diversity and altered community composition in an antibiotic- and concentration-dependent manner. Two antibiotic conditions were selected for pure-culture isolation, resulting in 74 isolates that represented 28 different species based on genomic average nucleotide identity. These include 13 putatively novel species based on GTDB classification, and 19 species belonging to nine globally abundant AS core genera. Although several isolates belonged to genera with cultured representatives, they likely represent distinct species with potentially different ecological functions and physiological traits. These findings demonstrate that antibiotics can function as ecological selectors during cultivation and aid the targeted isolation of ecosystem-relevant activated sludge bacteria.

**IMPORTANCE:**

Biological wastewater treatment relies on diverse microbial communities to degrade pollutants and drive nutrient transformations. Understanding the physiology and metabolism of these microorganisms is essential for improving the efficiency and cost-effectiveness of treatment processes. Much of our current knowledge is derived from 16S rRNA gene amplicon sequencing and metagenomic analyses. However, validating these sequencing- and genome-based insights requires bacterial species as pure cultures, and only a limited number of taxa common in wastewater treatment plants are currently available in culture. Here, we present an isolation strategy that uses antibiotics as a selective pressure to reduce microbial complexity and alleviate competitive exclusion during cultivation, while full-length 16S rRNA gene amplicon sequencing is used to monitor enrichment and guide targeted isolation, thereby facilitating the recovery of process-relevant activated sludge bacteria, including potentially uncultured taxa. These isolates can serve as model organisms for experimental validation of genome-based predictions.

## INTRODUCTION

Wastewater treatment plants (WWTPs) ensure that wastewater is cleaned and can be safely returned to the water cycle (1–3). Traditionally, the focus of WWTPs has been on ensuring the quality of the treated effluent. However, nutrient-rich wastewater also presents opportunities for recovery of resources such as phosphorus, nitrogen, and biopolymers (1, 4, 5). Resource recovery is tightly connected to the biological secondary treatment, and the most widely used technology for this is the activated sludge (AS) process. In this process, complex microbial communities growing as AS flocs assimilate or transform nutrients and degrade pollutants remaining in wastewater after primary treatment. These flocs contain diverse aggregates of bacteria, including floc-forming and filamentous microorganisms (2). Accordingly, the quality of the treated effluent and the efficiency of resource recovery depend on both the microbial composition of the AS and the operational conditions of the treatment system (1, 2, 4, 5).

While molecular methods, such as 16S rRNA gene amplicon sequencing and genome-resolved metagenomics, have greatly advanced our understanding of the microbial composition of AS and identified genes involved in key treatment processes, the specific roles of individual microbes within the system remain incompletely understood. Gaining deeper insights into these microorganisms is essential for improving control over WWTPs and facilitating the transition to more sustainable water resource recovery facilities (WRRFs) (6). Additionally, genome-based predictions of metabolic traits of AS microbes must be confirmed through controlled experiments in the laboratory (7). Consequently, obtaining pure cultures from AS is important for experimentally validating genome-based predictions and advancing our understanding of wastewater microbial ecology, ultimately supporting efforts to improve the efficiency and operation of WWTPs. Unfortunately, many abundant bacterial taxa in AS have never been isolated as pure cultures, supported by the fact that 46% (115/250) of the core genera identified in the MiDAS 4 global survey of WWTPs were assigned MiDAS placeholder or *Candidatus* taxonomies (2). The challenge in obtaining pure cultures from AS arises from the need to separate individual cells from the floc aggregates, the difficulty in replicating the specific environmental conditions required for the bacteria to grow, and the complex interdependencies between species essential for growing certain bacteria (8–10). In addition, some microorganisms may enter physiological states such as viable but non-culturable (VBNC) (11) or persister states (12, 13), which can further complicate recovery by limiting or delaying colony formation under standard cultivation conditions. Given the importance of having pure cultures available, there is a pressing need to improve existing methods for isolating microorganisms (14).

We have recently developed an isolation strategy that uses agarose plates containing a minimal growth medium supplemented with filter-sterilized AS fluid (ASF) to grow single cells from AS prepared using a combination of homogenization, sonication, and serial filtration (15). This approach aims to recreate a culture medium that closely resembles the oligotrophic conditions in which many AS microorganisms thrive. In that study, amplicon sequencing revealed that at least 76 previously uncultured species could be grown on ASF plates, and 10 of these were isolated as pure cultures. Despite these significant advancements, the approach did not fully address the challenge posed by fast-growing bacteria on the ASF agarose plates, which tended to outcompete slower-growing activated sludge bacteria. This issue is particularly problematic when both types of bacteria compete for the same substrate (7).

To address these challenges, it may be advantageous to reduce microbial community complexity to enrich for species of interest prior to isolation. We hypothesized that antibiotics could be used as selective agents to reduce community complexity during cultivation, thereby alleviating competitive exclusion and enabling the growth of target species (7). In support of this, previous research has shown that susceptibility to antibiotics can be influenced by physiological state and growth rate, with slow-growing or metabolically less active bacteria often exhibiting increased tolerance to antibiotic exposure (13, 16–18). The application of antibiotics has previously led to the successful isolation of 79 strains of Planctomycetes, based on the observation that members of this phylum are intrinsically resistant to certain antimicrobials, such as ampicillin, streptomycin, and carbenicillin (19). However, given the broad diversity of microbes in AS and the limited knowledge of antibiotic resistance in AS taxa, it remains challenging to use a single antibiotic to selectively inhibit unwanted bacterial growth.

In this study, we investigated whether the addition of antibiotics to ASF agarose plates could reduce the community complexity of colony-forming activated sludge bacteria by transiently suppressing subsets of fast-growing taxa. We evaluated 11 antibiotics from six different classes at three concentrations each (Table 1). 16S rRNA gene amplicon sequencing of biomass recovered from antibiotic-supplemented plates was used to identify taxa enriched under different antibiotic-mediated selection regimes, thereby informing strategies for the targeted isolation of key AS microorganisms.

**TABLE 1:** Antibiotics used and their concentrations^*a*^

Class	Antibiotic	Low	Medium	High
Aminoglycosides	Kanamycin (Sigma-Aldrich, K1377)	20	100	500
	Gentamicin (Sigma-Aldrich, G1397)	10	50	250
	Neomycin (Sigma-Aldrich, N1142)	40	200	1,000
	Streptomycin (Sigma-Aldrich S6501)	20	100	500
β-lactams	Ampicillin (Sigma-Aldrich, A9518)	40	200	1,000
	Carbenicillin (Fisher Scientific, BP2648)	20	100	500
Fluoroquinolones	Ciprofloxacin (Fluka, 17,850)	5	25	125
Macrolides	Erythromycin (Sigma-Aldrich, E5389)	20	100	500
Semisynthetic	Chloramphenicol (Sigma-Aldrich, C0378)	2	10	50
	Rifampicin (Calbiochem, 557,303)	10	50	250
Tetracyclines	Tetracycline (Sigma-Aldrich, T3258)	10	50	250

^*a*^ The antibiotic concentrations in µg/mL are expressed as either low, medium, or high concentrations relative to concentrations commonly used for *E. coli.*

## RESULTS AND DISCUSSION

### Antibiotics and incubation time influence colony formation

**Fig 1:**
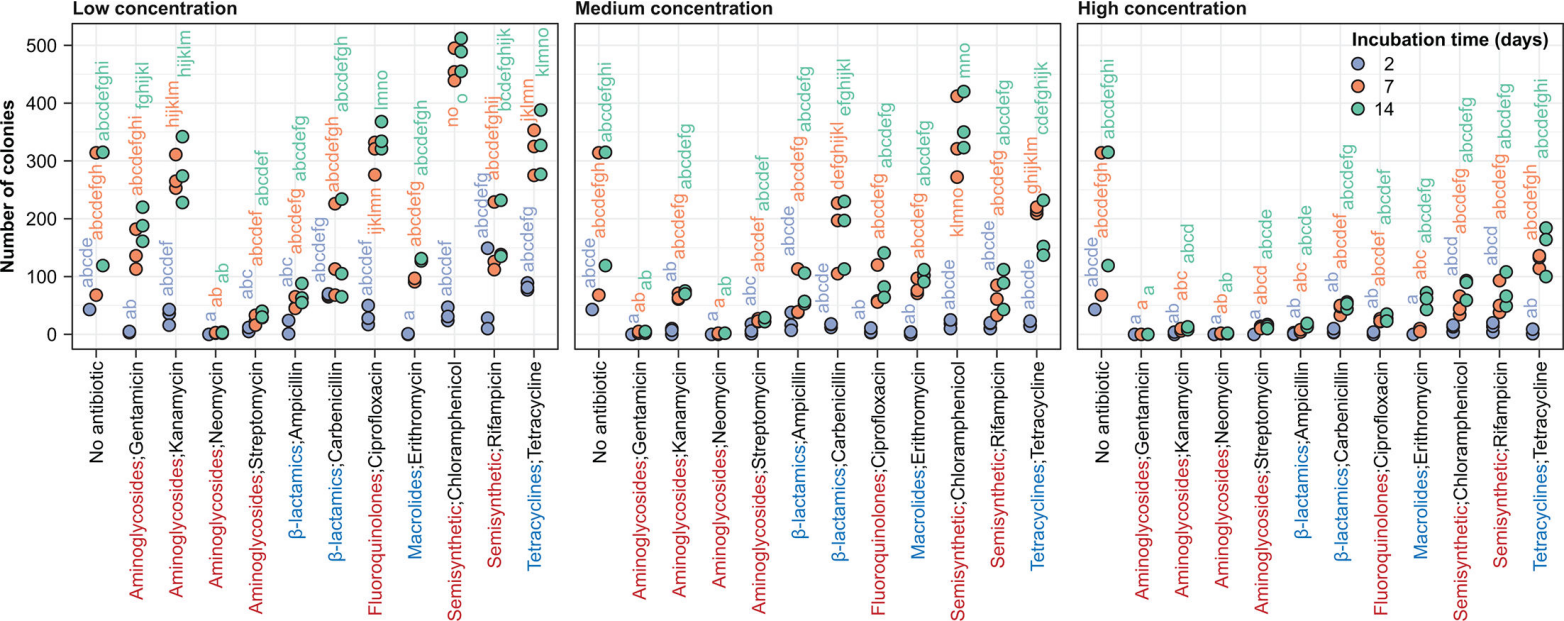
Colony counts on ASF agarose plates with antibiotics. The number of colonies was counted after 2, 7, and 14 days of bacterial growth on ASF agarose plates containing the different antibiotics at low, medium, or high concentration as specified in Table 1. The same “no antibiotic” controls were used in all plots. Differences in colony counts among antibiotic, concentration, and incubation-time combinations were assessed using one-way ANOVA followed by Tukey-adjusted pairwise comparisons (*α* = 0.05). Results are shown using compact letter display, where groups that do not share letters are significantly different.

To assess how antibiotic selection and incubation time influenced bacterial recovery from AS, we monitored colony formation on antibiotic-supplemented and control plates across concentrations and over time (Fig. 1). The number of colonies was strongly influenced by antibiotic concentration, with each antibiotic exhibiting a distinct effect. Overall, aminoglycosides had the strongest negative impact on colony numbers, resulting in significantly lower colony counts than most other treatments at medium and high concentrations, with neomycin showing the most pronounced effect across all concentrations. In contrast, plates containing chloramphenicol at low and medium concentrations, as well as kanamycin, ciprofloxacin, and tetracycline at low concentrations, showed colony numbers that were not significantly different from, or in some cases higher than, control plates without antibiotics.

After 2 days of incubation, colony numbers were generally low across all conditions. However, for most conditions, the colony numbers increased significantly by day 7 and showed only minor additional increases up to day 14. The delayed emergence of colonies was most pronounced for plates supplemented with bacteriostatic antibiotics such as tetracycline, chloramphenicol, and erythromycin, whereas plates containing bactericidal antibiotics showed little to no late colony formation, particularly at high concentrations (Fig. 1).

Together, these patterns suggest that at least two processes contribute to the observed dynamics: the presence of intrinsically slow-growing taxa and the transient suppression of fast-growing populations under bacteriostatic conditions. In addition, delayed colony formation may also reflect the recovery of persister cells or bacteria transitioning from VBNC-like physiological states following transient antibiotic exposure (11–13). Consistent with the contribution of slow-growing organisms, similar increases during prolonged incubation were also observed on control plates without antibiotics (Fig. 1; Fig. S1). In addition, the recovery of growth on bacteriostatic antibiotic plates is likely facilitated by declining antibiotic activity over time, further reducing selective pressure during extended incubation. Transient inhibition of fast-growing populations may also reduce competitive interactions, thereby allowing otherwise suppressed taxa to emerge.

### Antibiotics reduce microbial diversity

To assess the effects of antibiotic treatment and incubation on microbial diversity, full-length 16S rRNA gene amplicon sequencing was performed on DNA extracted from the total microbial biomass scraped from individual agarose plates after 14 days of growth, as well as from the source AS. To minimize bias due to uneven sequencing depth, all samples were rarefied to 5,000 reads, after which 66 plate samples were retained for downstream analyses.

**Fig 2:**
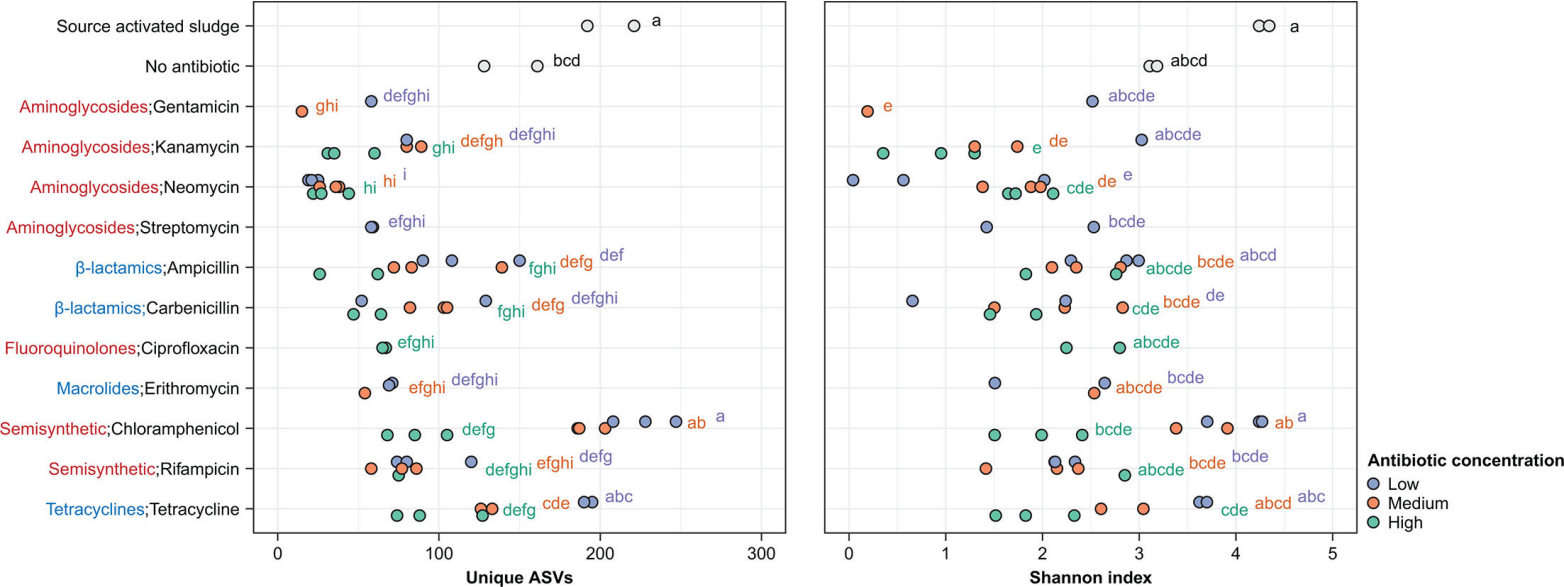
Alpha-diversity of biomass collected from ASF agarose plates with and without antibiotics. Biomass for microbial profiling was collected from ASF agarose plates after 14 days of growth. Alpha-diversity was expressed as the number of unique ASVs and the Shannon diversity index. The source activated sludge was included as a control. Differences among treatments were assessed using one-way ANOVA followed by Tukey-adjusted pairwise comparisons (*α* = 0.05). Results are shown using compact letter display, where groups that do not share letters are significantly different.

The total number of observed amplicon sequence variants (ASVs) and the Shannon diversity index were significantly reduced on almost all antibiotic-supplemented plates when compared to both the source AS and plates without antibiotics (Fig. 2). The lowest diversity was observed on plates with the aminoglycosides: gentamicin, kanamycin, and neomycin, and the effect was more pronounced when higher concentrations of the antibiotics were used. In contrast, we only observed a reduction in diversity on plates with chloramphenicol and tetracycline when these were supplied at high concentrations. Low concentrations of these antibiotics were associated with increased diversity compared to the plates without antibiotics, although this effect was not significant. These effects of the antibiotics on alpha diversity were in line with the previously observed effects on colony formation (Fig. 1). Indeed, a strong correlation (*r* = 0.89, *P* < 2.2 × 10^−16^) was found between the number of colonies and the number of unique ASVs (Fig. S2). This increased alpha diversity on plates with low concentrations of chloramphenicol and tetracycline likely reflects reduced competitive exclusion following partial suppression of fast-growing taxa, although contributions from intrinsic tolerance or antibiotic resistance cannot be excluded (7).

### Antibiotics alter microbial community composition

**Fig 3:**
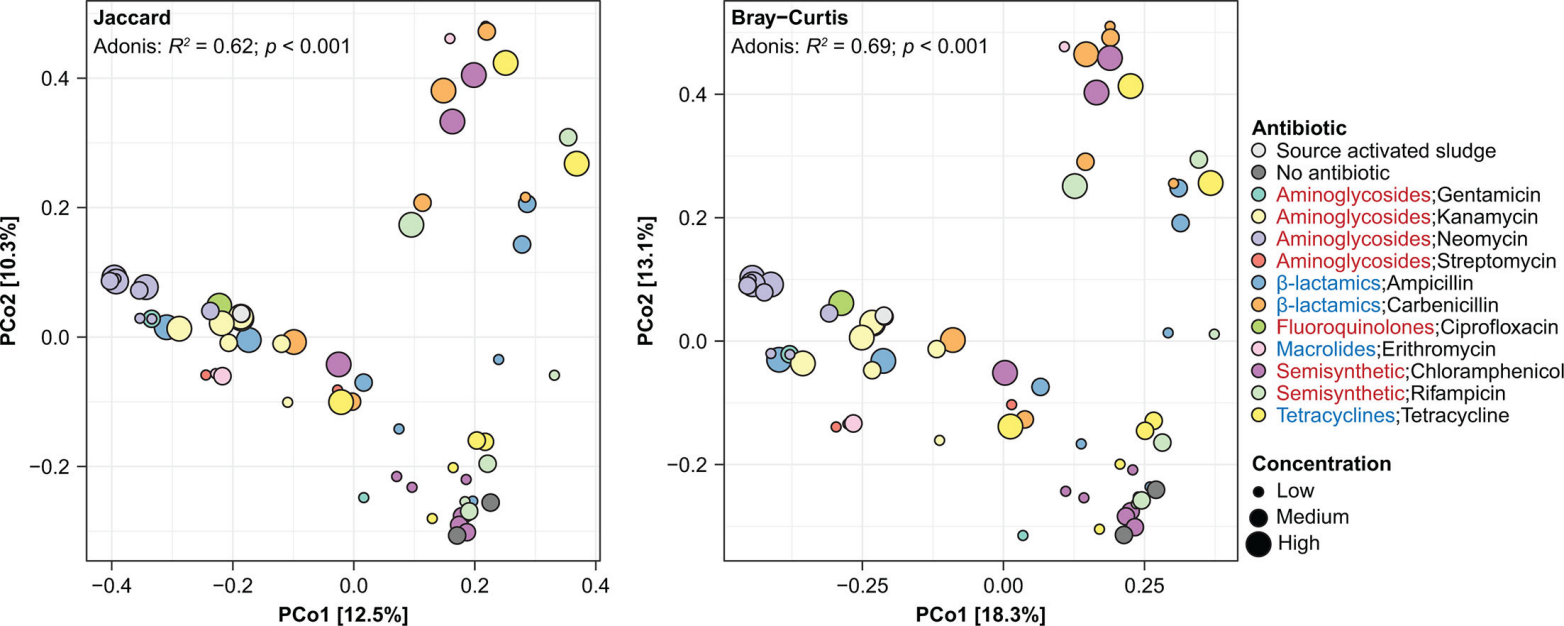
Beta-diversity of biomass collected from ASF agarose plates with and without antibiotics. Principal coordinate analysis (PCoA) plots are based on Jaccard (presence/absence) and Bray-Curtis (relative abundance) distance measures at the ASV-level. The proportion of variation in the microbial community explained by each antibiotic in different concentrations was determined by a permutational multivariate analysis of variance (PERMANOVA, Adonis R^2^ values).

To evaluate how microbial community composition varied among plates containing different antibiotics, principal coordinate analysis (PCoA) was performed (Fig. 3). This analysis showed that communities grown on plates without antibiotics were more similar to those grown with chloramphenicol and tetracycline at low and medium concentrations, whereas communities on ampicillin-, kanamycin-, and ciprofloxacin-supplemented plates were more similar to the source AS. A PERMANOVA analysis showed that a large proportion of the variance among samples could be accounted for by the combination of antibiotic identity and concentration (*R*² = 0.69, *P* < 0.001), whereas antibiotic identity alone (*R*² = 0.44, *P* < 0.001) or antibiotic class alone (*R*² = 0.29, *P* < 0.001) explained a smaller proportion of the variance. This indicates that both the specific antibiotic and its concentration were important determinants of community composition on the plates.

**Fig 4:**
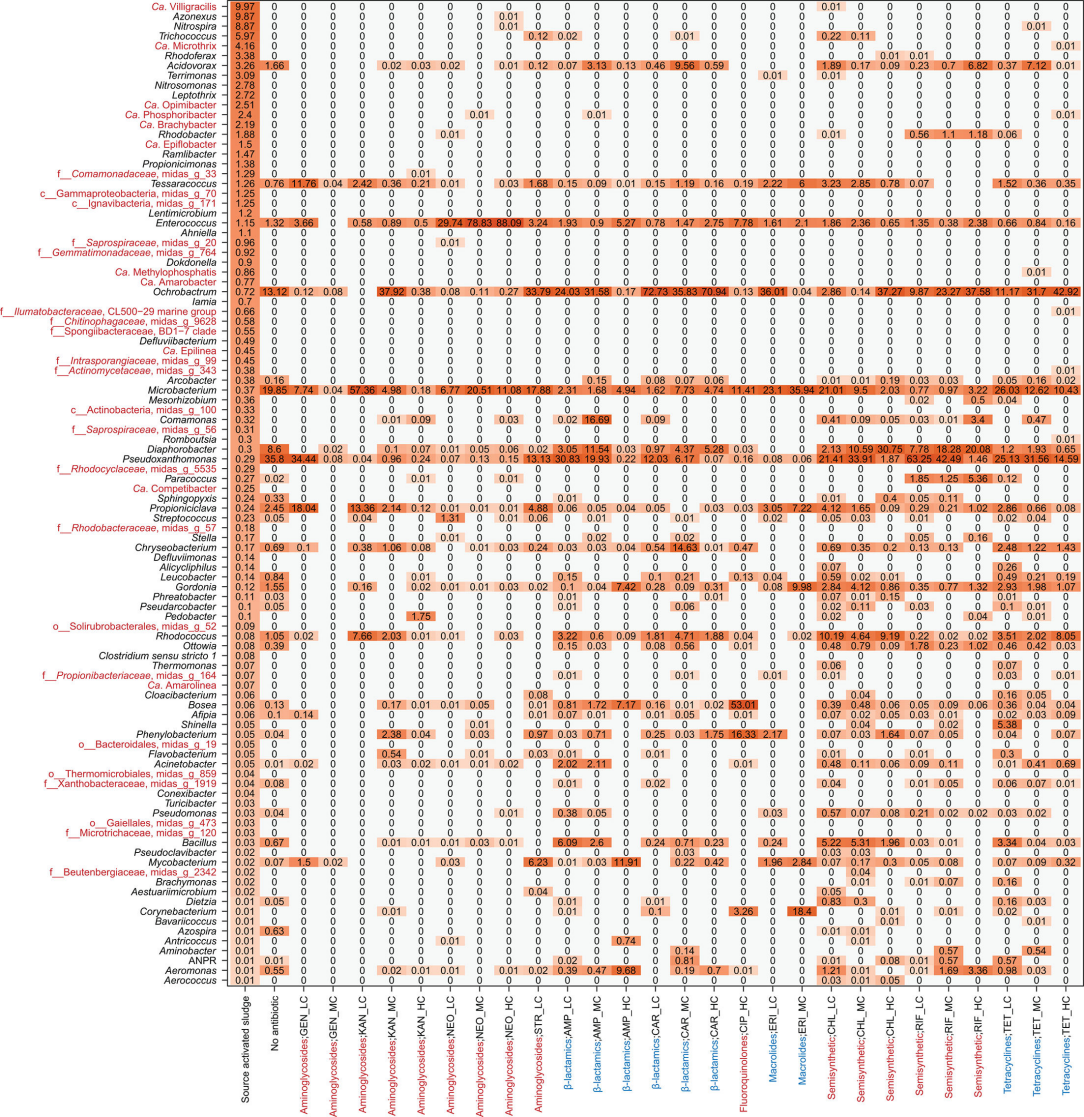
Heatmap of the top 100 most abundant genera found in AS from Aalborg West WWTP and their relative abundances on each agarose plate. Values show the mean of replicates. Names in red correspond to genera without a cultured representative. For genera assigned MiDAS placeholder names, the lowest available officially described taxonomic rank is also shown. AMP: ampicillin, CAR: carbenicillin, GEN: gentamicin, KAN: kanamycin, NEO: neomycin, STR: streptomycin, CHL: chloramphenicol, RIF: rifampicin, CIP: ciprofloxacin, ERI: erythromycin, TET: tetracycline, ANPR: *Allorhizobium−Neorhizobium−Pararhizobium−Rhizobium,* LC: low concentration, MC: medium concentration, HC: high concentration.

To determine which bacterial taxa had grown on the different ASF plates, the amplicon data were classified using the MiDAS 5.3 database, which provides full-length 16S rRNA gene sequences with a complete seven-level taxonomy (domain to species) for most bacteria and archaea present in wastewater treatment systems and anaerobic digesters (20, 21). Out of the 100 most abundant genera found in the source AS, 33 correspond to candidate genera or to genera identified only by MiDAS placeholder names, which likely represent previously uncultured taxa (Fig. 4). However, all these genera were present only at low relative abundance on the ASF plates. The placeholder genera midas_g_1919 (family *Xanthobacteraceae*), midas_g_2342 (family *Beutenbergiaceae*), and midas_g_164 (family *Propionibacteriaceae*) were the most abundant, representing up to 0.08%, 0.04%, and 0.03% relative abundance, respectively. In addition, seven uncultured genera were detected by only a single read in the rarefied amplicon data.

The consistently low relative abundance of uncultured genera suggests that these taxa represent only a limited fraction of the biomass produced during plate growth. This interpretation is supported by the pronounced differences in colony size observed on the plates (Fig. S1), where a small number of fast-growing taxa formed large colonies, while many other taxa produced only small colonies even after 14 days of incubation. In addition, many slow-growing bacteria harbor fewer 16S rRNA gene copies than fast-growing taxa (22, 23), which may further reduce the observed relative abundance of previously uncultured genera in amplicon-based analyses.

These observations indicate that the current cultivation strategy may be insufficient to support robust growth of many previously uncultured genera present in the source AS. This highlights the challenge of replicating the environmental conditions required to cultivate many uncultured taxa. Furthermore, some microorganisms may no longer be viable after being dispersed from flocs or may require co-dependent interactions with one or more other microorganisms (7). Likewise, oxygen requirements can be difficult to predict or emulate, although recent advances have been made in this area (24). Establishing such conditions for each genus of interest would likely be time- and resource-demanding. One possible continuation of this work would be to prepare solid media with reduced nutrient supplementation, or to rely solely on the nutrients present in the ASF, while using community-resolved amplicon profiling to assess whether these conditions enrich ecologically relevant activated sludge taxa. This approach would aim to further enrich slow-growing bacteria that may be adapted to low-substrate conditions (25).

To further support the novelty of the growing bacteria, the ASVs from the plates were compared to the Life Tree Project (LTP) database from August 2023, which contains 16S rRNA gene sequences from bacteria and archaea that have previously been isolated (26). The novelty of the ASVs was determined by the percent identity shared with the closest relative in the LTP database and the taxonomic thresholds provided by Yarza et al. (27). Based on these thresholds, 36 of the 100 most abundant ASVs represented previously uncultured species (identity 94.5-98.7%), 24 new genera (identity 81.0-94.5%), and nine new families (identity <81.0%) (Fig. S3).

### Assessment of novelty and cultivation success

To cultivate isolates, dispersed cells from AS were spread on ASF plates supplemented with either tetracycline at medium concentration or rifampicin at high concentration, because these conditions allowed the growth of several abundant and process-relevant AS genera (Fig. 4). After two weeks of growth, 84 colonies from the tetracycline plates and 12 from the rifampicin plates were picked, re-streaked onto fresh plates, and DNA was extracted for genome sequencing from the resulting colonies. Genome assembly of the 96 colonies resulted in 74 isolate genomes, representing 28 different species according to genome dereplication at 95% average nucleotide identity (ANI).

To assess the taxonomic novelty and ecological relevance of the recovered isolates, we evaluated their classification across multiple reference databases. The novelty of the isolated species was first determined by assigning taxonomy to the recovered genomes using the Genome Taxonomy Database (GTDB) release 220 (Table S1). Thirteen of the 28 dereplicated genomes were either unclassified at the species level or assigned alphanumeric placeholder names, suggesting that they represent previously uncultured species. However, when we mapped the 16S rRNA genes from the genomes to the LTP database, only three shared <98.7% identity with their best hit, indicative of novel species (Table S2). This discrepancy likely reflects the limited species-level resolution of 16S rRNA gene similarity compared to genome-based taxonomy, particularly for taxa with conserved 16S rRNA genes (28, 29). Finally, we examined whether any of the isolates corresponded to the core or conditionally rare or abundant taxa (CRAT) defined in the MiDAS global survey of WWTPs (2) (Table S3). Interestingly, 19 out of 28 isolated species could be assigned to MiDAS AS core genera (9× *Microbacterium*, 3× *Gordonia*, 2× *Ottowia*, *Acidovorax*, *Mycobacterium*, *Propioniciclava*, *Streptococcus*, and *Thauera*) based on 16S rRNA gene identity >94.5%, and one was classified as a CRAT genus (*Rhodococcus*). This indicates that most isolates belong to microbial groups that are consistently abundant and associated with key wastewater treatment processes, rather than representing only rare or opportunistic taxa. These results indicate that the applied isolation strategy can facilitate the recovery of ecologically important bacteria from activated sludge.

### Growth of diverse genera under antibiotic-mediated selection

Microbial profiling of biomass scraped from ASF plates provided insight into which bacterial genera were able to grow under antibiotic-mediated selection. Importantly, growth on antibiotic-supplemented plates does not necessarily reflect phenotypic resistance, particularly given the prolonged incubation without further antibiotic supplementation. Instead, growth under these conditions likely reflects a combination of intrinsic tolerance, slow growth rates, and reduced competition due to suppression of some fast-growing taxa, as previously observed for bacteria exposed to antibiotic stress under non-lethal or static conditions (30).

To explore taxon-specific growth patterns, we compared the genera detected on plates without antibiotics to those detected on plates containing antibiotics (Fig. S4). Across all conditions, a broad range of genera were able to grow in the presence of antibiotics. Growth was most frequently observed on plates containing chloramphenicol, rifampicin, and tetracycline, even at high concentrations, whereas fewer genera were detected on plates supplemented with aminoglycosides and β-lactams. These patterns are consistent with the distinct modes of action of the antibiotics tested (bacteriostatic vs bactericidal) and their differential impacts on fast- and slow-growing bacteria, as well as with previous observations that slow-growing taxa are often less susceptible to growth inhibition by antibiotics than rapidly dividing organisms (7).

**Fig 5:**
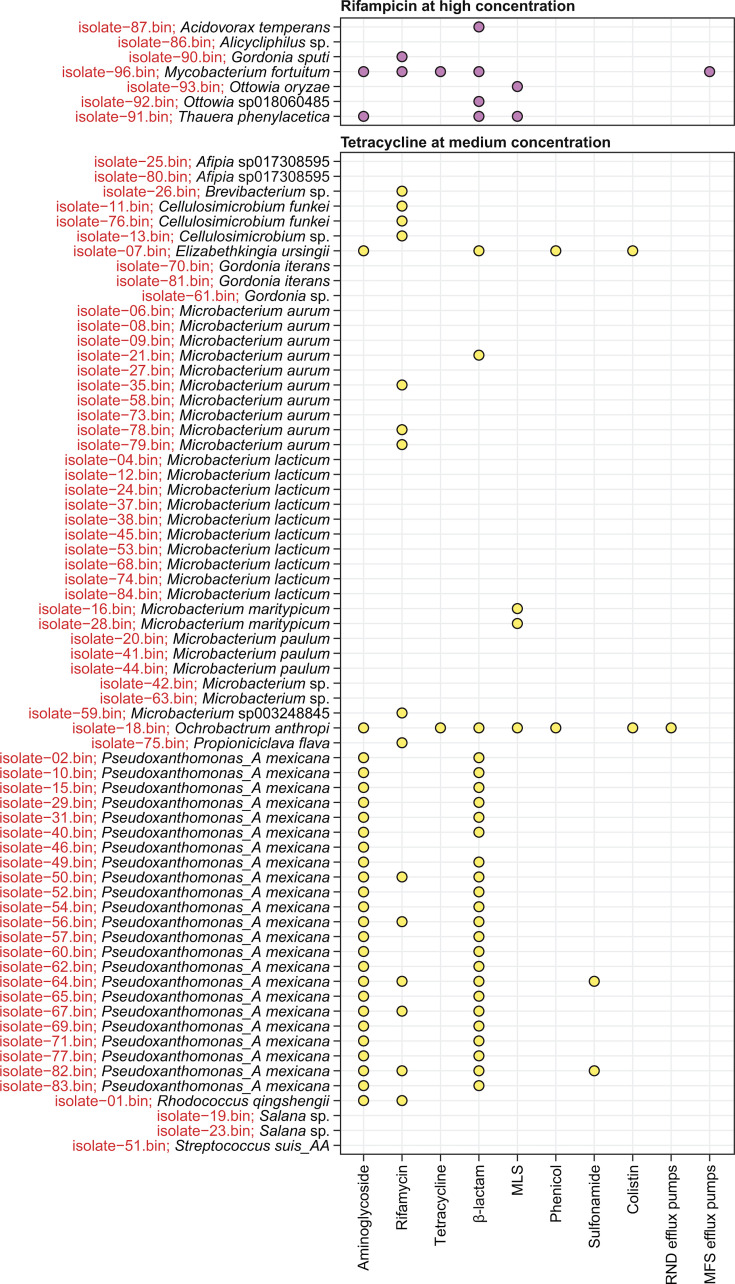
Resistance gene categories detected in the genomes of the 74 isolates recovered from plates with rifampicin and tetracycline. Each row represents a single isolate genome, labeled by isolate ID and taxonomic assignment, and each column represents a resistance category detected using a stringent SARG + homology screen (DIAMOND blastx; ≥90% amino acid identity and ≥90% subject coverage). Dots indicate the presence of at least one gene belonging to the corresponding resistance category. Efflux-associated categories (RND and MFS) represent transporter families with broad substrate ranges and are included to illustrate intrinsic tolerance mechanisms rather than antibiotic-specific resistance. MLS, macrolide-lincosamide-streptogramin.

To further assess whether growth under antibiotic selection could be explained by known resistance mechanisms, we compared growth patterns with genome-based resistance gene annotations for the pure-culture isolates (Fig. 5). Canonical resistance genes corresponding to the selecting antibiotic were detected in only a small fraction of the isolates. Among the 64 isolates recovered from plates containing a medium concentration of tetracycline, tetracycline resistance genes were identified in only a single genome, while rifamycin resistance genes, which provide resistance to rifampicin, were detected in only two of the seven isolates obtained from plates with a high concentration of rifampicin. These discrepancies indicate that growth under antibiotic selection is frequently not explained by the presence of annotated resistance genes. Instead, these results highlight the limited predictive power of current resistance gene databases for environmental bacteria and underscore that antibiotic-mediated selection during cultivation can favor taxa through mechanisms other than canonical resistance, including intrinsic tolerance, physiological state, or slow growth (7, 31, 32). In this context, antibiotics functioned primarily as a selective pressure to reduce competitive exclusion rather than as a definitive screen for resistance phenotypes.

Together, these findings support the use of antibiotics as a tool to modulate community composition during cultivation, enabling the recovery of diverse and ecologically relevant activated sludge taxa that would otherwise be outcompeted under non-selective conditions.

### Conclusion and perspectives

In summary, the addition of antibiotics to ASF-based agarose plates successfully reduced microbial diversity and altered community composition, often in ways that favored the growth of slow-growing and ecologically relevant activated sludge bacteria. Accordingly, the present study should be viewed primarily as a proof-of-concept demonstration of antibiotic-mediated ecological selection as a cultivation strategy for AS bacteria. Although the cultivation conditions may not have been fully optimized to maximize the recovery of novel genera, the approach showed promise for isolating previously uncultured species, including several species belonging to the core microbiome of AS systems.

Importantly, the ability to selectively isolate and culture ecosystem-relevant bacteria, especially those involved in nutrient removal and resource recovery, opens new avenues for downstream research in synthetic biology and microbiome engineering. These cultured strains can serve as model organisms for validating genome-based predictions, understanding microbial interactions, and enhancing desired traits. Future studies should evaluate the reproducibility and effectiveness of antibiotic-mediated selection across AS systems operating under different conditions and containing distinct microbial community compositions. More broadly, the use of antibiotics as ecological selectors during cultivation may also be applicable to other complex microbial ecosystems where competitive exclusion limits the recovery of slow-growing or low-abundance taxa.

## MATERIALS AND METHODS

### Preparation of ASF and growth media

Five liters of AS were collected from the aeration tank at Aalborg West WWTP on 7 December 2023. Three liters of AS were filtered through a coffee filter placed in a funnel and then filter-sterilized using a Nalgene 0.1 µm bottle-top sterile filter (Thermo Fisher Scientific). The obtained ASF was stored at 4°C until used.

A concentrated stock of Reasoner’s 2A (R2A) medium was prepared as follows: 50 g/L of yeast extract (MP Biomedicals, #103303), 30 g/L of sodium pyruvate, 50 g/L of proteose peptone (Bacto, Thermo Fisher Scientific, #211693), 5 g/L of K_2_HPO_4_, and 50 g/L of MgSO_4_·7H_2_O were dissolved in demineralized water and filter-sterilized as above.

A concentrated stock of a carbon and nitrogen (C/N) source was prepared with 5 g/L of casamino acids (Amresco Inc, #J851), 10 g/L of tryptone (Fluka, #70937), 12.5 g/L of sodium acetate, and 5.3 g/L of NH_4_Cl that were added to demineralized water and filter sterilized.

For solid media, 250 mL of 10 g/L agarose (Electran Agarose DNA Pure Grade) was autoclaved at 121°C for 20 min. Then, 730 mL of ASF, pre-heated to 60°C, was added along with 10 mL of concentrated R2A and 10 mL of concentrated C/N source.

For 1 L of liquid medium, 10 mL of concentrated R2A and 10 mL of concentrated C/N source were added to 980 mL of ASF.

Eleven antibiotics were selected and added to the medium at low, medium, and high concentrations in triplicate (see Table 1). The medium concentration was based on the standard working concentration of each antibiotic for *E. coli*, while the low and high concentrations corresponded to one-fifth and five times the medium concentration, respectively.

### Preparation of dispersed single cells from AS

To disperse cells from AS flocs, 30 mL of AS was homogenized using a RZR 2020 benchtop stirrer with a Teflon tissue grinder attached for 1 min in second gear. Five mL of the homogenized AS was sonicated using a Bandelin Sonopuls HD2200 with an MS73 probe, set at an amplitude of 60% with six pulses of 10 s with intervals of 10 s and immediately kept on ice. The solution was filtered through a 40 µm cell strainer (VWR) and centrifuged at 3,000 × *g* for 5 min and further filtered through a 5 µm cell strainer (pluriSelect Life Science) and centrifuged at 8,600 × *g* for 2 min. Cells in the permeate were counted in a Bürker-Türk chamber and diluted to 10,000 cells/mL with sterile-filtered ASF.

### Bacterial growth on agarose plates

One hundred microliters of the diluted cell suspension was spread on ASF agarose plates with an antibiotic at low, medium, or high concentrations in triplicate. In addition, three plates without antibiotics were used as controls. The plates were sealed with parafilm and incubated at 25°C for at least 14 days. Plates were photographed at 2, 7, and 14 days of incubation to monitor the number and appearance of colonies using an imaging device created based on the protocol by Smith and Schuster (33).

### DNA extraction

DNA was extracted from biomass scraped off agarose plates and from three replicates of the source AS using the FastDNA Spin kit for soil (MP Biomedicals) following the MiDAS protocol for plate extraction (aau_wwtp_dna_v_8.0) available at https://www.midasfieldguide.org/guide/protocols. The concentration of extracted DNA was measured with the Qubit 1× dsDNA assay (Thermo Fisher Scientific) in a Tecan Infinite 200 PRO plate reader.

### 16S rRNA gene amplicon sequencing

The V1–V8 region of the 16S rRNA gene was amplified and barcoded for PacBio sequencing. Each PCR reaction contained 12.5 µL of 2× KAPA HiFi HotStart ReadyMix, 3 µL of 2.5 µM barcoded 27F forward (5′-GCATC/barcode/AGRGTTYGATYMTGGCTCAG-3′) primer (34), 3 µL of 2.5 µM 1391R reverse (5′-GCATC/barcode/GACGGGCGGTGWGTRCA-3′) primer (35), 5 µL of 0.5 ng/µL template DNA, and 1.5 µL of nuclease-free water. PCR conditions were 95°C for 3 min, followed by 25 cycles of denaturation at 95°C for 30 s, annealing at 57°C for 30 s, and elongation at 72°C for 1 min.

The PCR products were purified using 0.8× AMPure XP DNA beads (Agencourt) and eluted in 25 µL nuclease-free water. Concentration of the amplicons was measured with the Qubit 1× dsDNA assay, and the size of the amplicons was confirmed using an Agilent TapeStation HS 5000 screen tape. Seven µL of each barcoded sample were pooled and stored at −20°C until sequencing.

The multiplexed amplicons were prepared for PacBio sequencing using the SMRTbell Prep Kit 3.0, then sequenced on a Revio SMRT Cell 25M using a 24-h movie and an on-plate loading concentration of 200–300 pM on a PacBio Revio instrument.

### Data analysis

Demultiplexing of sequenced reads was performed using PacBio’s package Lima v.2.9.0, following the pipeline for a HiFi run from a FASTQ file with asymmetric barcodes lima --hifi-preset ASYMMETRIC. Demultiplexed reads were oriented against the SILVA v138.1 SSURef NR99 ARB database (36) using usearch v11 (37) with the usearch -orient command. Primer sequences were removed using cutadapt v4.6 (38) with the command cutadapt -g ^AGRGTTYGATYMTGGCTCAG...TGYACWCACCGCCCGTC --discard-untrimmed. Quality filtering was performed with the usearch -fastq_filter -fastq_maxee 1.0 command. Unique sequences were identified with usearch -fastx_uniques, and ASVs were resolved with the usearch -unoise3 -zotus. The ASV table was generated with usearch -otutab with the -zotus and -strand plus option. Taxonomic classification of the obtained ASVs was performed with the MiDAS 5.3 database (20) and SINTAX (39) with the usearch -sintax -sintax_cutoff 0.8 -strand plus command. In addition, the obtained ASVs were mapped against the Life Tree Project (LTP) database from August 2023 (26) using the command usearch -usearch_global -maxrejects 0 -maxaccepts 0 -top_hit_only. ASVs associated with new lineages were determined based on the sequence identity with the closest relative in the LTP database and the thresholds of <98.7% for new species, <94.5% for new genera, and <86.5% for new families (27).

All plots and statistical analyses were performed in R v4.5.0 (40) through RStudio v2025.05.0 (41) using the packages tidyverse v2.0.0 (42), ggplot2 v3.5.2 (43), ampvis2 v2.8.7 (44), vegan v2.6-4 (45), multcomp v1.4-28 (46), multcompView v0.1-10 (47), and emmeans v1.11.2 (48).

### Isolation of pure cultures

For isolation, 5 L of AS was collected from the aeration tank at Aalborg West on 5 September 2024. A 30 mL portion of the sample was used for cell dispersion, while the remaining volume was used to prepare ASF for agarose plates as previously described. Rifampicin and tetracycline were added at high and medium concentrations, respectively. Each plate was inoculated with approximately 1,000 cells, sealed with parafilm, and incubated at 25°C for 2 weeks. A total of 96 colonies were randomly selected from the plates, re-streaked on fresh ASF agarose plates with the corresponding antibiotic, and incubated at 25°C for at least 7 days or until visible colony formation. Several colonies were scraped off each plate and resuspended in 200 µL of liquid medium, from which 160 µL were used for DNA extraction as previously described. The remaining 40 µL was transferred to cryotubes containing 100 µL of 40% glycerol solution for storage at −80°C.

### Genome sequencing of isolates

Extracted DNA from the isolates was normalized to a concentration of 20 ng/µL in a final volume of 10 µL. Samples that did not achieve this concentration were instead used in their entirety in a final volume of 10 µL. All samples were then barcoded using the Nanopore Rapid Barcoding Kit 96 v14 protocol, pooled, and prepared for sequencing on the PromethION platform with a FLO-PRO114M flow cell. The reads were basecalled with Dorado v7.4.13 using the super-accuracy model.

### Genome assembly, classification, and identification of antibiotic resistance genes

Sequencing reads were processed using filtlong v.0.2.1, discarding the worst 10% and any reads shorter than 1,000 bp. Whole-genome assemblies were generated with nanophase v0.2.3 (49), initially using the isolate module. For assemblies exhibiting contamination levels above 10%, determined by checkM2, the assembly process was repeated using the meta module. Assembled genomes with a completeness below 70% were excluded from further analysis. Taxonomic classification was carried out with the Genome Taxonomy Database Toolkit v2.4.0 (GTDB-Tk) (50) using the --classify_wf command and the --skip_ani_screen argument. In addition, the 16S rRNA gene sequences were extracted from the whole genomes and mapped against the LTP 08_2023 database to further test the novelty of the species (26), and against the MiDAS 4.8 database (2) to identify core and CRAT genera. Antibiotic resistance genes in the genomes were identified by matching open reading frames against the SARG+ database release (2025-07-21) (51) using diamond blastx with the following settings: minimum 90% identity, minimum 90% subject coverage, and an *e*-value cutoff of 1e−25.

## Supplementary Material

Reviewer comments

## Data Availability

The raw sequencing data and genomes generated in this study have been deposited in the European Nucleotide Archive (ENA) at EMBL-EBI under accession number PRJEB85528.

## SUPPLEMENTAL MATERIAL

Supplemental figures spectrum.01477-26-s0001.pdf Fig. S1 to S4.

Supplemental tables spectrum.01477-26-s0002.xlsx Tables S1 to S3.

OPEN PEER REVIEW reviewer-comments.pdf An accounting of the reviewer comments and feedback.

ASM does not own the copyrights to Supplemental Material that may be linked to, or accessed through, an article. The authors have granted ASM a non-exclusive, world-wide license to publish the Supplemental Material files. Please contact the corresponding author directly for reuse.
