# Supplementary material for: Application of antibiotics for the selective isolation of previously uncultured species from activated sludge
Source: Microbiol Spectr. 2026 Jun 22;14(8):e01477-26. doi: 10.1128/spectrum.01477-26 (PMC13436025; doi:10.1128/spectrum.01477-26)
Supplement: Supplementary file 2 [file spectrum.01477-26-s0001.pdf]

Supplementary Information for:

Application of antibiotics for the selective isolation of previously uncultured bacteria from activated sludge

Authors: Stefania Andrea Rosso Villanelo,[a] Sofie Zacho Vestergaard,[a] Lei Liu,[a] Yu Yang,[a] Inge Søkilde Pedersen,[b,c] Per Halkjær Nielsen,[a] and Morten Kam Dahl Dueholm[a]#

Affiliations:
[a]Center for Microbial Communities, Department of Chemistry and Bioscience, Aalborg University, Aalborg, Denmark.
[b]Department of Clinical Medicine, Aalborg University, Aalborg, Denmark.
[c]Department of Molecular Diagnostics, Aalborg University Hospital, Aalborg, Denmark.

[#]Correspondence to: Morten Kam Dahl Dueholm, Center for Microbial Communities, Department of Chemistry and Bioscience, Aalborg University, Fredrik Bajers Vej 7H, 9220 Aalborg, Denmark; Phone: +45 9940 8508; Fax: Not available; Email: md@bio.aau.dk

Table of contents:

Page 2-4: Figure S1-S4

Supplementary Figures:

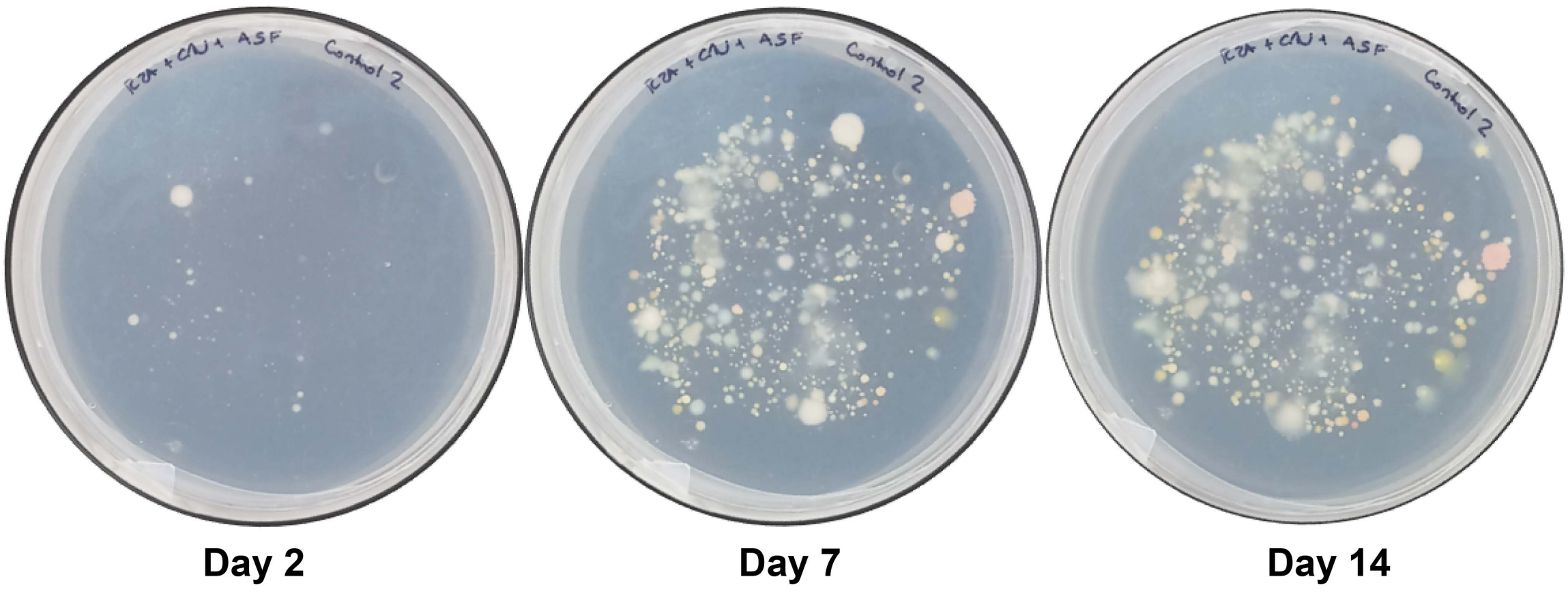

Figure S1. Photographs of one of the ASF plates without antibiotics after 2, 7, and 14 days of growth.

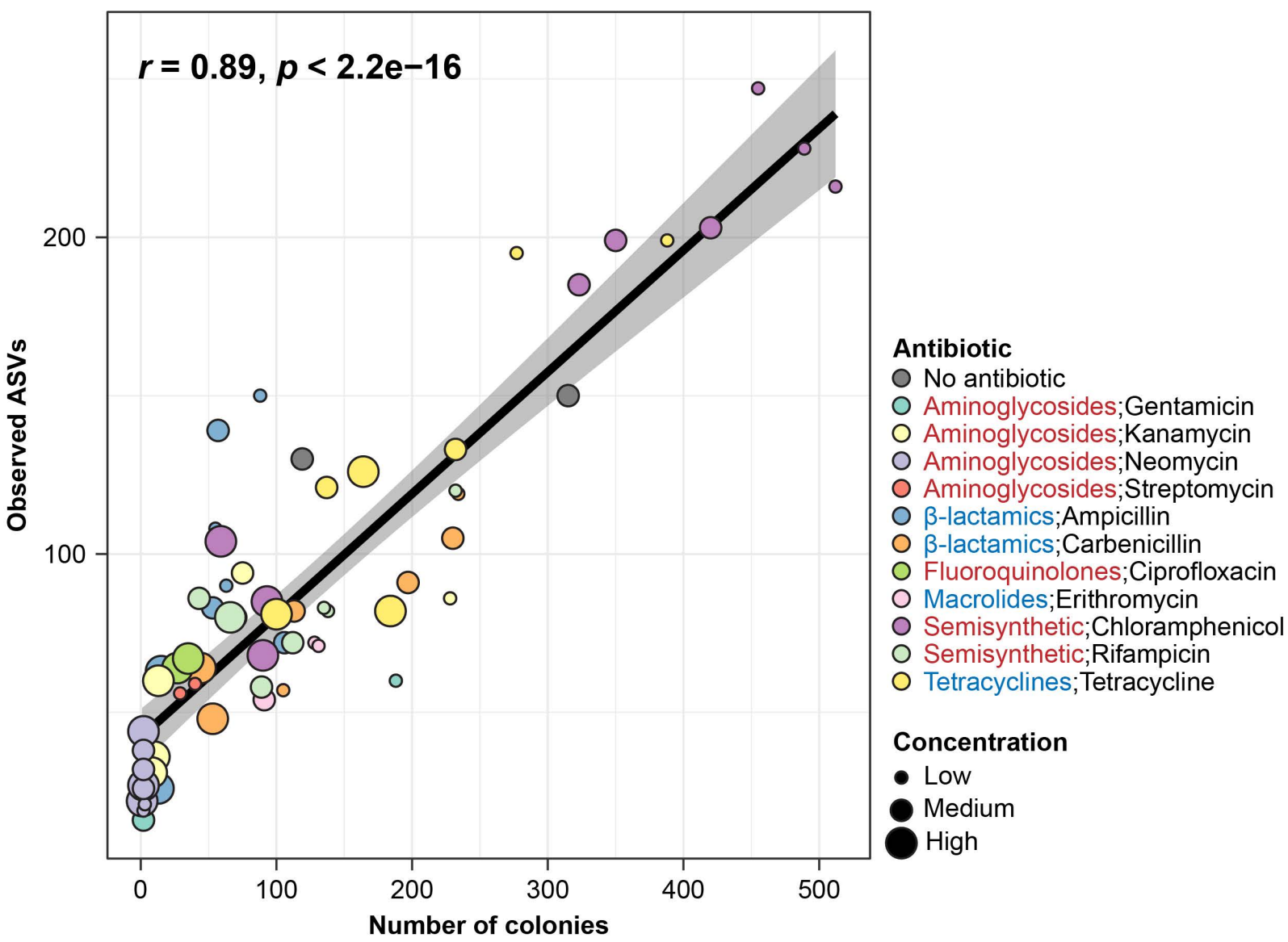

Figure S2: Correlation (Pearson) between the number of colonies counted on each plate after 14 days and the number of observed ASVs.

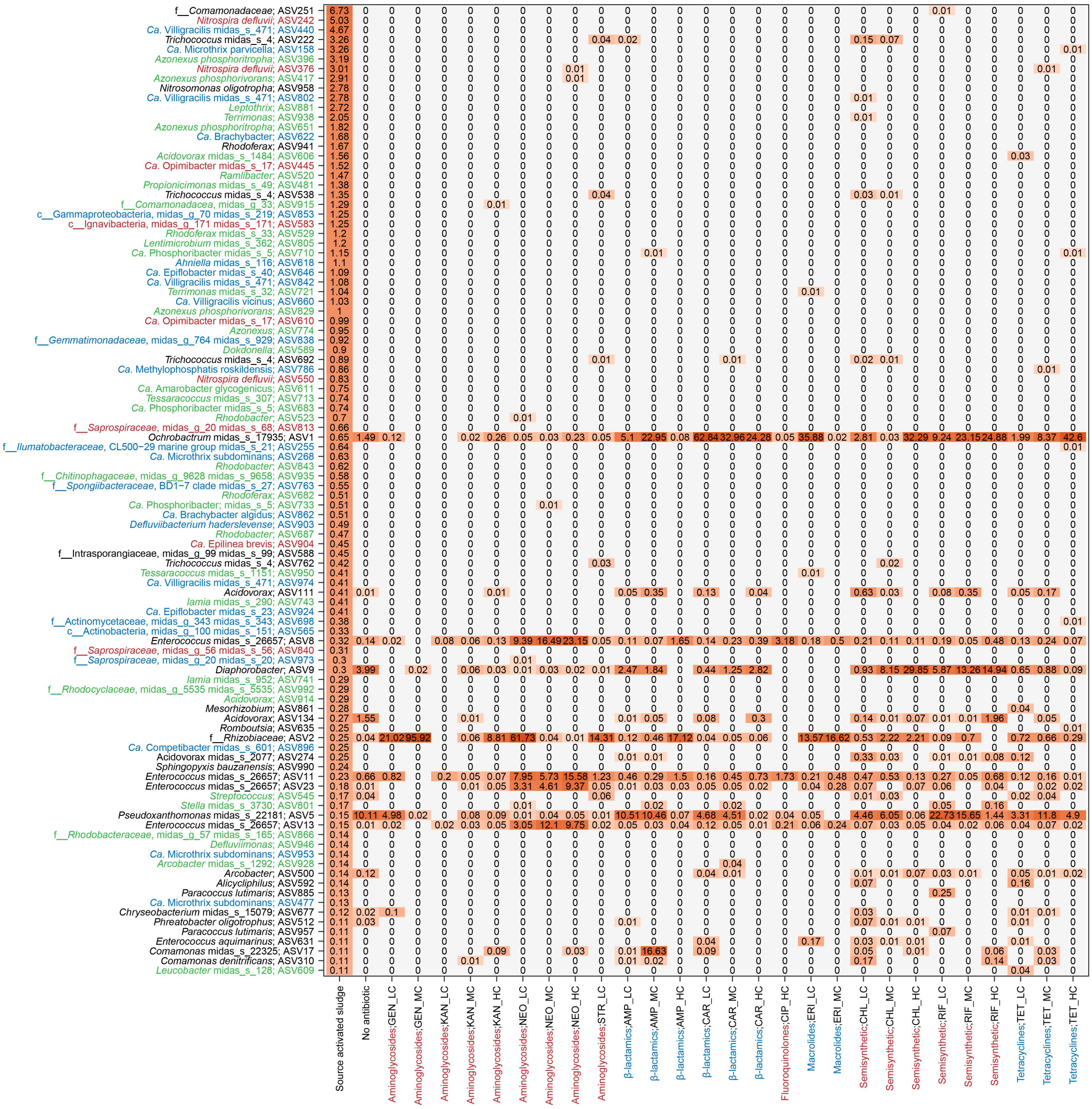

Figure S3. Heatmap of the top 100 most abundant ASVs found in the source AS and in the biomass collected from ASF agarose plates with various antibiotics. For ASVs classified with placeholder names, the lowest available officially described taxonomic rank is also shown. The values represent the average percent relative abundance across replicates. The colors of the ASVs and their assigned taxonomy indicate how close the ASV is to its closest match in the LTP database. Black: known species (>98.7%); green: new species (94.5-98.7%); blue: new genera (81.0-94.5%); red: new families (<81.0%). AMP: ampicillin, CAR: carbenicillin, GEN: gentamicin, KAN: kanamycin, NEO: neomycin, STR: streptomycin, CHL: chloramphenicol, RIF: rifampicin, CIP: ciprofloxacin, ERI: erythromycin, TET: tetracycline, LC: Low concentration, MC: Medium concentration, HC: High concentration.

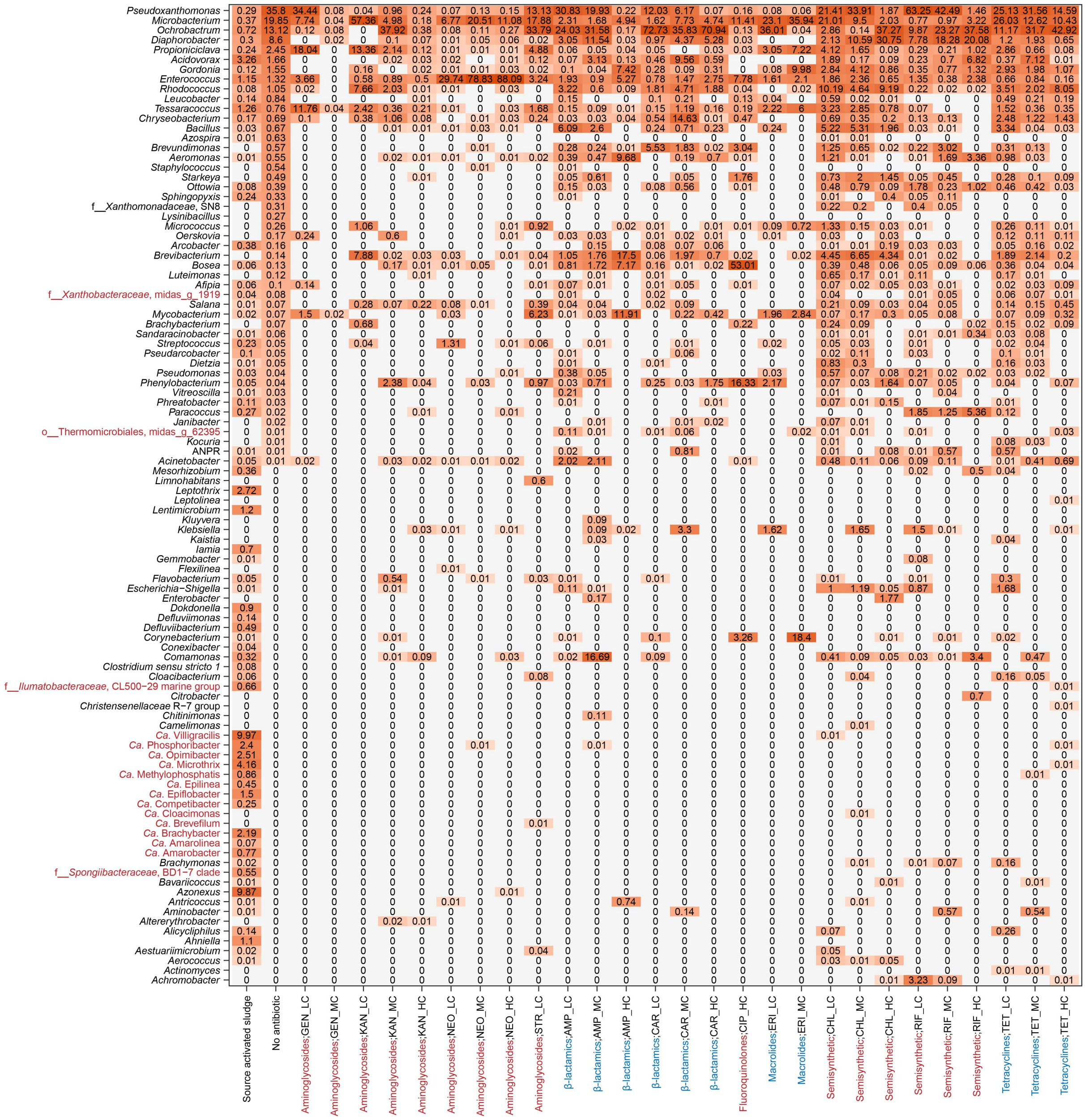

Figure S4. Heatmap of the 100 most abundant genera found in the source AS and in the biomass collected from ASF agarose plates with various antibiotics. For genera assigned placeholder names, the lowest available officially described taxonomic rank is also shown. The heatmap is sorted based on the plates without antibiotics. Names in red correspond to genera that do not have a cultured representative. The values represent the average percent relative abundance across replicates. AMP: ampicillin, CAR: carbenicillin, GEN: gentamicin, KAN: kanamycin, NEO: neomycin, STR: streptomycin, CHL: chloramphenicol, RIF: rifampicin, CIP: ciprofloxacin, ERI: erythromycin, TET: tetracycline, LC: Low concentration, MC: Medium concentration, HC: High concentration ANPR: *Allorhizobium−Neorhizobium−Pararhizobium−Rhizobium*.
